# Advancing Our Understanding of Executive Functioning Development—Measurements and Promotion in Naturalistic Contexts

**DOI:** 10.3390/brainsci15060621

**Published:** 2025-06-09

**Authors:** Andrea Paula Goldin, Marcos Luis Pietto, Juan Esteban Kamienkowski

**Affiliations:** 1 Universidad Torcuato Di Tella, Escuela de Negocios, Centro de Inteligencia Artificial y Neurociencia (CIAN), Laboratorio de Neurociencia, Av. Figueroa Alcorta 7350, Buenos Aires C1428BCW, Argentina; 2 Consejo Nacional de Investigaciones Científicas y Técnicas (CONICET), Buenos Aires C1425FQD, Argentina; 3 Centro de Educación Médica e Investigaciones Clínicas “Norberto Quirno” (CEMIC-CONICET), Unidad de Neurobiología Aplicada (UNA), Buenos Aires C1431FWO, Argentina; 4 Universidad Nacional de Hurlingham (UNAHUR-CONICET), Instituto de Educación, Hurlingham B1688GEZ, Argentina; 5 Laboratorio de Inteligencia Artificial Aplicada (LIAA), Instituto de Ciencias de la Computación, Facultad de Ciencias Exactas y Naturales, Universidad de Buenos Aires, CONICET, Buenos Aires C1428EGA, Argentina; 6 Universidad de Buenos Aires, Facultad de Ciencias Exactas y Naturales, Departamento de Computación, Buenos Aires C1428EGA, Argentina; 7 Universidad de Buenos Aires, Maestría de Explotación de Datos y Descubrimiento del Conocimiento, Buenos Aires C1428EGA, Argentina

The collection of eight articles featured in this Special Issue of *Brain Sciences* highlights the multifaceted nature of executive functioning (EF), spanning early childhood to adulthood and encompassing diverse contexts—including typical development, clinical populations, and various cultural settings. Together, these contributions underscore the growing importance of studying EF in ecologically valid ways and exploring interventions that can be seamlessly integrated into everyday life.

This Issue approaches EF from different angles: from assessments conducted in natural settings and targeted interventions for children, to broader investigations in diverse populations and critical discussions around cultural and methodological validity. Most of the articles focus on understanding, assessing, or improving executive functions in children or adults, with measurements as diverse as from babies and stroke patients.

**Investigating EF Through Development and Neuroscience**

Building on our understanding of EF, Pietto et al. examined error monitoring in preschoolers from low socioeconomic backgrounds using a go/no-go task and electroencephalography (EEG). Their findings suggest that children can adjust their behavior adaptively following errors, potentially linked to error-related theta activity—offering insight into the neurophysiological underpinnings of early performance monitoring, a core component of EF.

Further exploring the neural basis of EF, Zeng et al. examined whether upregulating a small-world brain network via functional near infrared spectroscopy (fNIRS) neurofeedback could improve inhibitory control. They observed changes in the topological properties of the brain network, and a correlation between increased small-worldness and improved inhibition, suggesting that targeted neurofeedback may be a promising avenue for enhancing EF.

**Linking EF to Daily Functioning**

Several articles explore how EF relates to daily functioning. Sakai et al. categorized stroke patients into groups based on the severity of executive dysfunction and compared their walking abilities, finding that greater executive dysfunction—measured via the Trail Making Test Part B—was associated with poorer walking ability. This research highlights the critical role of higher-order cognitive functions in supporting everyday activities in a clinical population.

Thellung di Courtelary et al. used n-back tasks and questionnaires to evaluate how mood, stress, and psychological factors influence working memory and apathy in healthy adults. While their results did not show a direct impact of mood on working memory performance, they found that phobic anxiety negatively predicted numerical working memory, and that anxiety and emotion dysregulation were linked to increased task withdrawal. These findings contribute to a more nuanced understanding of the emotional and motivational underpinnings of EF. 

Using data from the Millennium Cohort Study, Zhou and Tolmie analyzed the longitudinal associations between early motor skills and later EF and academic achievement. Their results emphasize the importance of both gross and fine motor skills in shaping cognitive development (measured by the Cambridge Neuropsychological Test Battery, CANTAB), underscoring the value of incorporating motor activities into the educational curricula.

**Promoting EF Through Naturalistic Interventions**

Other articles focus on promoting EF through naturalistic interventions. Souza et al. reported a pilot study on a school-based intervention that embedded EF training within daily activities in 8-to-10-year-olds with executive dysfunction complaints (EF was evaluated through the Inventory of Executive Functioning Difficulties, Regulation, and Delay Aversion for Children). Focusing on metacognition, the program showed modest but promising improvements in working memory and inhibition, as reported by both tests and observations from parents and teachers. 

Interestingly, Eng et al. explored the feasibility of using gamified tasks and machine learning to assess EF in preschoolers. By comparing gamified and traditional versions of the Eriksen Flanker Task for assessing selective attention, they demonstrated the potential for engaging digital tools in early EF assessment, while also addressing methodological considerations in adapting and applying cognitive tasks for young children. Similarly, Vladisauskas et al. [1] assessed EF remotely in typical classrooms using a battery of computerized games over eight years across three countries. Their findings support the reliability of remote, classroom-based EF assessments and reveal clear developmental trends across ages.

**Cultural Considerations in EF Assessment**

Several contributions to this Special Issue emphasize the value of conducting EF research outside laboratory settings, moving toward research that better reflects real-life functioning. Jukes et al. critically examined how EF assessments must be adapted for cultural contexts. They argue that EF tasks developed in one context often carry cultural assumptions that do not hold in different cultural settings and call for a systematic approach to ensuring ecological validity in diverse populations elsewhere. Their discussion underscores how cultural norms regarding motivation, social interaction, reasoning, familiarity, and time perception can affect test performance and the universality of EF constructs.

This issue features authors affiliated with institutions across Argentina, Australia, Brazil, China, Italy, Japan, South Africa, the United Kingdom, and the United States—demonstrating the global interest in EF research. However, as Jukes et al. note, most EF studies still originate from Western, Educated, Industrialized, Rich, and Democratic (WEIRD) populations. While the geographic diversity of contributors marks a step toward broader representation, it also reinforces the urgent need for more cross-cultural research.

As findings from psychological research, largely based on WEIRD populations, cannot be assumed to be universally applicable e.g., in [2], cross-cultural research provides the opportunity to test the generality and ecological validity of existing EF theories and measures beyond WEIRD populations. Jukes et al. emphasize that cultural preferences related to motivation, social interaction, thinking styles, and concepts of time can significantly impact performance on EF tasks. Cross-cultural research is essential for identifying these culturally specific factors and for adapting or developing new assessment tools that are ecologically valid and reliable across diverse populations.

Findings from WEIRD populations cannot be assumed to apply universally. Cross-cultural studies are essential for testing the generalizability of EF theories and identifying culturally specific influences. By distinguishing between universal cognitive processes and culture-specific manifestations of EF, such research can inform more inclusive theories and tools. It also opens pathways for developing interventions tailored to diverse educational and cultural contexts—contributing to more equitable and effective educational practices globally.

The contributions to this Special Issue, with their multinational authorship, signal a shift towards a more global perspective on EF development. Embracing and expanding cross-cultural research offers significant opportunities to refine our understanding of EF, develop more equitable assessment practices, and design culturally sensitive interventions that benefit children and adults worldwide.

**Conclusions**

The studies presented in this Special Issue demonstrate the progress of the field in understanding, assessing, and promoting executive functions across life stages and sociocultural contexts. Employing methodologies ranging from EEG and fNIRS to school-based interventions and gamified digital tools, these contributions collectively call for a paradigm shift in EF research and practice. Specifically, they advocate for:

- Developing ecologically valid assessment tools that are engaging, culturally sensitive, and reflective of how EF operates in daily life.
- Implementing naturalistic interventions embedded within existing routines, increasing the likelihood of transfer and granting sustainability.
- Recognizing the interplay of cognitive, motor, and socio-emotional development in shaping EF trajectories.
- Expanding research efforts to include diverse populations, clinical groups, and non-WEIRD cultural settings.

In sum, this Special Issue offers a compelling snapshot of current research in EF development. By highlighting innovative methodologies, the potential of naturalistic interventions, and the critical role of cultural and contextual factors, the articles herein provide valuable insights for researchers, educators, and clinicians alike on how EF skills function, and can be fostered to support positive developmental outcomes across a lifespan. The findings presented here also chart a path forward—one that emphasizes real-world relevance, cross-cultural inclusivity, and the practical promotion of executive skills in the rich complexity of real-world settings.

## References

[1] Vladisauskas M., Paz G.O., Nin V., Guillén J.C., Belloli L., Delgado H., Miguel M.A., Macario Cabral D., Shalom D.E., Forés A. (2024). The Long and Winding Road to Real-Life Experiments: Remote Assessment of Executive Functions with Computerized Games—Results from 8 Years of Naturalistic Interventions. Brain Sci..

[2] Jones D. (2010). A WEIRD View of Human Nature Skews Psychologists’ Studies. Science.

